# Risk of sleep disorders in patients with pneumoconiosis: a retrospective cohort study

**DOI:** 10.1007/s44197-024-00225-5

**Published:** 2024-04-04

**Authors:** Yen-Sung Lin, Te-Chun Shen, Cheng-Li Lin, Chih-Yen Tu, Te-Chun Hsia, Wu-Huei Hsu, Der-Yang Cho

**Affiliations:** 1 Division of Pulmonary and Critical Care Medicine, An Nan Hospital, China Medical University, Tainan, 709 Taiwan; 2 Department of Nursing, Chung Hwa University of Medical Technology, Tainan, 717 Taiwan; 3 Division of Pulmonary and Critical Care Medicine, Department of Internal Medicine, China Medical University Hospital, No. 2 Yu-De Road, Taichung, 404 Taiwan; 4 School of Medicine, College of Medicine, China Medical University, Taichung, 404 Taiwan; 5 Division of Critical Care Medicine, Chu Shang Show Chwan Hospital, Nantou, 557 Taiwan; 6 Management Office for Health Data, China Medical University Hospital, Taichung, 404 Taiwan; 7 Department of Neurosurgery, China Medical University Hospital, Taichung, 404 Taiwan

**Keywords:** Pneumoconiosis, Interstitial Lung Disease (ILD), Occupational disease, Sleep disorder, Sleep apnea

## Abstract

**Background:**

Pneumoconiosis is associated with pulmonary and cardiovascular diseases; however, the link between pneumoconiosis and sleep disorders is not well understood. This study aimed to investigate the connection between pneumoconiosis and subsequent risk of sleep disorders.

**Methods:**

This population-based retrospective cohort study used data from the National Health Insurance database in Taiwan. The pneumoconiosis cohort consisted of 13,329 patients newly diagnosed between 2000 and 2015. The comparison group included 53,316 age-, sex-, and diagnosis date-matched individuals without pneumoconiosis. The development of sleep disorders was monitored until the end of 2018. Cox proportional hazard regression models were used for risk assessment.

**Results:**

The incidence of sleep disorders was 1.31 times higher in the pneumoconiosis cohort than in the comparison cohort (22.8 vs. 16.2 per 1000 person-years). After controlling for age, sex, comorbidity, and medication, the adjusted hazard ratio (aHR) was 1.24 (95% confidence interval [CI] = 1.17–1.32). Stratified analyses by age group, sex, and comorbidity status showed significant associations between pneumoconiosis and sleep disorders (aHRs, 1.19–1.64). In addition, patients with pneumoconiosis had a significantly increased risk of developing sleep apnea (aHR = 1.71, 95% CI = 1.31–2.22).

**Conclusion:**

This study demonstrates that patients with pneumoconiosis are at a higher risk of developing sleep disorders and sleep apnea. Healthcare professionals should pay close attention to sleep quality and disturbances in patients with pneumoconiosis.

**Supplementary Information:**

The online version contains supplementary material available at 10.1007/s44197-024-00225-5.

## Introduction

Pneumoconiosis is a group of interstitial lung diseases (ILD) caused by inhaling mineral dust or fiber and is the most common occupational disease. Workers usually come into contact with these harmful substances in their workplace environment [1]. Despite its preventable nature, pneumoconiosis remains prevalent, particularly in developing countries, with over 500,000 living cases and 60,000 new cases reported annually [2]. The disease carries a high mortality rate, with over 20,000 deaths reported annually [3]. Inhaling mineral dust or fiber can lead to various respiratory and cardiovascular disorders, including progressive massive fibrosis, pneumothorax, chronic obstructive pulmonary disease (COPD), atrial fibrillation, congestive heart failure (CHF), coronary artery disease (CAD), peripheral arterial disease, and cerebrovascular disease (CVD) [4–11], which often contribute to premature death [12–14].

Pneumoconiosis may be associated with sleep disorders as a result of combination of respiratory abnormality and alterations in sleep architecture. Chronic cough and breathlessness could contribute to sleep disturbance [15–18]. Interstitial changes and poor pulmonary function, leading to decreased oxygen saturation, could also decrease sleep efficiency [19, 20]. In addition, patients with pneumoconiosis experienced increased stage 1 sleep, decreased slow-wave sleep, decreased rapid eye movement sleep, increased sleep fragmentation, and increased arousal [19, 20].

Evidence showed that sleep disturbance was prevalent in patients with pneumoconiosis. Several studies confirmed that patients with pneumoconiosis have poorer sleep quality than those without it [15, 16, 21–23]. However, previous studies have been limited by their small size, study design, and follow-up duration. Thus, this research aimed to address these limitations using a nationwide, population-based, retrospective cohort study to explore the association between pneumoconiosis and subsequent risk of sleep disorders.

## Materials and methods

### Data Sources

The National Health Insurance (NHI) program in Taiwan, which covered over 99.5% of its residents since 2010, maintains a comprehensive database managed by the Ministry of Health and Welfare. This database containing the total population in the NHI system between 1998 and 2018, which contains extensive medical data such as demographic information, clinical visit dates, diagnostic codes, prescriptions, and treatments, was used in this study. Data were analyzed in the Health and Welfare Data Science Center, Taiwan Ministry of Health and Welfare (at China Medical University), and the access permit number was H109068. This study was approved by the Research Ethics Committee of China Medical University Hospital (CMUH107-REC2-181).

### Study cohorts

**Fig. 1:**
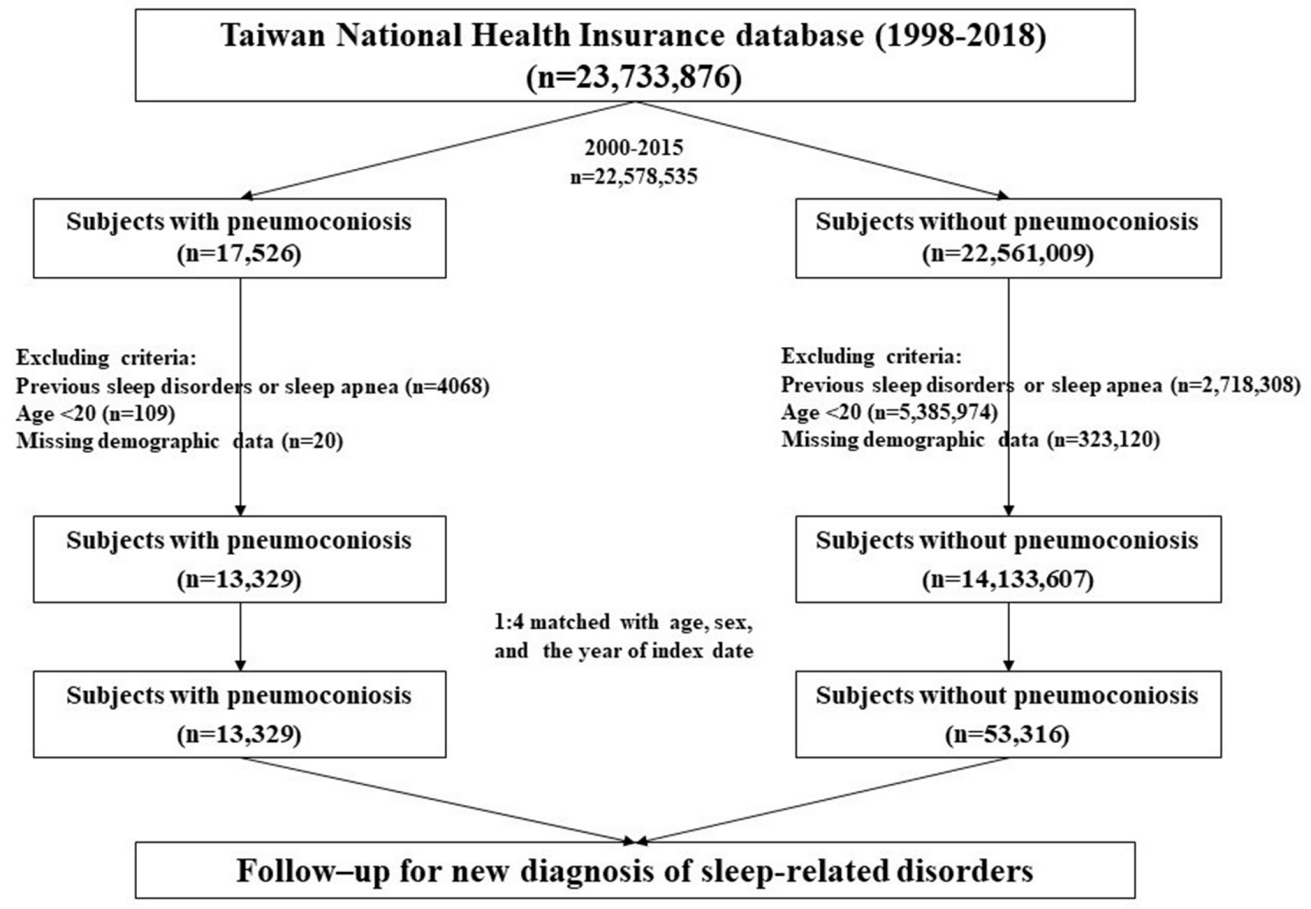
Flowchart of study participants selection

In Taiwan, Occupational Safety and Health Administration of the Ministry of Labor (http://www.osha.gov.tw) had published official guidelines to diagnose pneumoconiosis (supplementary data) based on International Labour Organization International Classification of Radiographs of Pneumoconiosis (https://www.ilo.org), which had updated in 1980, 2000 and 2011. The study enrolled adult patients newly diagnosed with pneumoconiosis (International Classification of Diseases [ICD] codes 500–505 and J60–J65) between 2000 and 2015 in the pneumoconiosis cohort (Fig. 1). Pneumoconiosis was diagnosed when the ICD code was registered in one inpatient discharge record or two outpatient visit records [8, 24, 25]. Using ICD codes for defining diseases in the Taiwan NHI database has been validated, and most of the reported positive predictive values ranged from 80 to 99% [26]. The date of pneumoconiosis diagnosis was defined as the index date, we had excluded those with a previous diagnosis of pneumoconiosis from January 1, 1998, to the index date. Patients with preexisting sleep disorders were excluded. The comparison cohort consisted of adults without pneumoconiosis, 1:4 matching with age, sex, and the index date. Both groups were monitored until the diagnosis of sleep disorders/sleep apnea, insurance system withdrawal, death, or end of 2018.

### Outcomes and covariates

The primary outcomes were sleep-related disorders (ICD codes 307.4 [specific disorders of sleep of nonorganic origin], 327 [organic sleep disorders], 780.5 [sleep disturbances], F51 [sleep disorders not due to a substance or known physiological condition], and G47 [sleep disorders]), which were classified as sleep apnea (SA, ICD codes 327.2, 780.51, 780.53, 780.57, and G47.3) and sleep disorders (other ICD codes from sleep-related disorders). We used accompanied prescription (Anatomical Therapeutic Chemical Classification System code: N05B and N05C) to validate sleep disorders and accompanied polysomnography (17008B) to confirm SA. Covariates such as age, sex, comorbidities, and medication were considered. Baseline comorbidities including hypertension (ICD codes 401–405 and I10–I16), diabetes mellitus (ICD codes 250 and E08–E13), hyperlipidemia (ICD codes 272 and E78), CVD (ICD 430–438 and I60–I69), asthma/COPD (ICD codes 491, 492, 493, 496, and J41–J45), chronic liver disease and cirrhosis (CLD, ICD codes 571 and K70–K77), chronic kidney disease (CKD, ICD codes 585 and N18), tuberculosis (ICD codes 010–018 and A15–A19), obesity (ICD codes 278 and E66), tobacco use disorders (ICD codes 305.1, 989.84, V15.82, F17, O99.33, P96.81, T65.2, Z57.31, Z71.6, Z72.0, Z77.22, and Z87.891), which may be associated with development of sleep-related disorders. Corticosteroid use (Anatomical Therapeutic Chemical Classification System code: H02) was defined as those using the medication for > 28 days.

### Statistical analysis

The chi-squared and Student t-tests were used to compare age groups, sex, comorbidities, and medication. The Kaplan–Meier method was used to estimate the cumulative incidence of sleep disorders in both cohorts, with the log-rank test determining significance. The study used univariate and multivariate Cox proportional hazard regression models to estimate crude and adjusted hazard ratios (cHRs and aHRs) and 95% confidence intervals (CIs). SAS statistical software (version 9.4 for Windows; SAS Institute, Inc., Cary, NC, USA) was used for data analysis, and a *p*-value of < 0.05 was deemed statistically significant.

## Results

A total of 13,329 patients with pneumoconiosis and 53,316 individuals without pneumoconiosis in a comparison cohort were included (Table 1). The mean age values were 70.2 ± 10.9 years and 70.1 ± 11.2 years in the pneumoconiosis and comparison cohorts, respectively, in which 91.4% of individuals in both cohorts were male. The proportions of comorbidities and medication use in the two cohorts were as follows: hypertension, 51.8% vs. 52.3%; diabetes mellitus, 7.07% vs. 4.00%; hyperlipidemia, 20.1% vs. 24.6%; CVD, 15.8% vs. 12.1%; asthma/COPD, 49.9% vs. 12.7%; CLD 12.9% vs. 8.23%; CKD, 3.52% vs. 1.69%; tuberculosis, 3.3% vs. 1.8%; obesity, 0.18% vs. 0.25%; tobacco use disorders, 1.19% vs. 0.65%; and corticosteroid use, 44.7% vs. 31.3%. No significant difference in hypertension and obesity was noticed between the two cohorts. The mean follow-up times were 5.61 ± 5.23 and 9.45 ± 5.25 years in the pneumoconiosis and comparison cohorts, respectively.

**Table 1:** Characteristics for individuals with and without pneumoconiosis

	Pneumoconiosis				
	No		Yes		
	*N* = 53,316		*N* = 13,329		
	n	%	n	%	*p*-value ^†^
Age					0.99
20 − 49	2664	5.0	666	5.0	
50 − 64	10,472	19.6	2618	19.6	
≥ 65	40,180	75.4	10,045	75.4	
Mean ± SD	70.1	± 11.2	70.2	± 10.9	0.20
Gender					0.99
Women	4604	8.6	1151	8.6	
Men	48,712	91.4	12,178	91.4	
Comorbidity					
Hypertension	27,864	52.3	6898	51.8	0.29
Diabetes mellitus	2131	4.0	943	7.1	< 0.001
Hyperlipidemia	13,088	24.6	2683	20.1	< 0.001
CVD	6472	12.1	2104	15.8	0.003
Asthma/COPD	6743	12.7	6655	49.9	< 0.001
CLD	4390	8.2	1720	12.9	< 0.001
CKD	902	1.7	469	3.5	< 0.001
Tuberculosis	960	1.8	446	3.3	< 0.001
Obesity	135	0.25	24	0.18	0.12
Tobacco use disorders	344	0.65	159	1.19	< 0.001
Medication					
Corticosteroid	16,705	31.3	5954	44.7	< 0.001

CKD, chronic kidney disease; CLD, chronic liver disease and cirrhosis; COPD, chronic obstructive pulmonary disease; CVD, cerebrovascular disease; SD, standard deviation

^†^ Chi-squired test and t-test

**Fig. 2:**
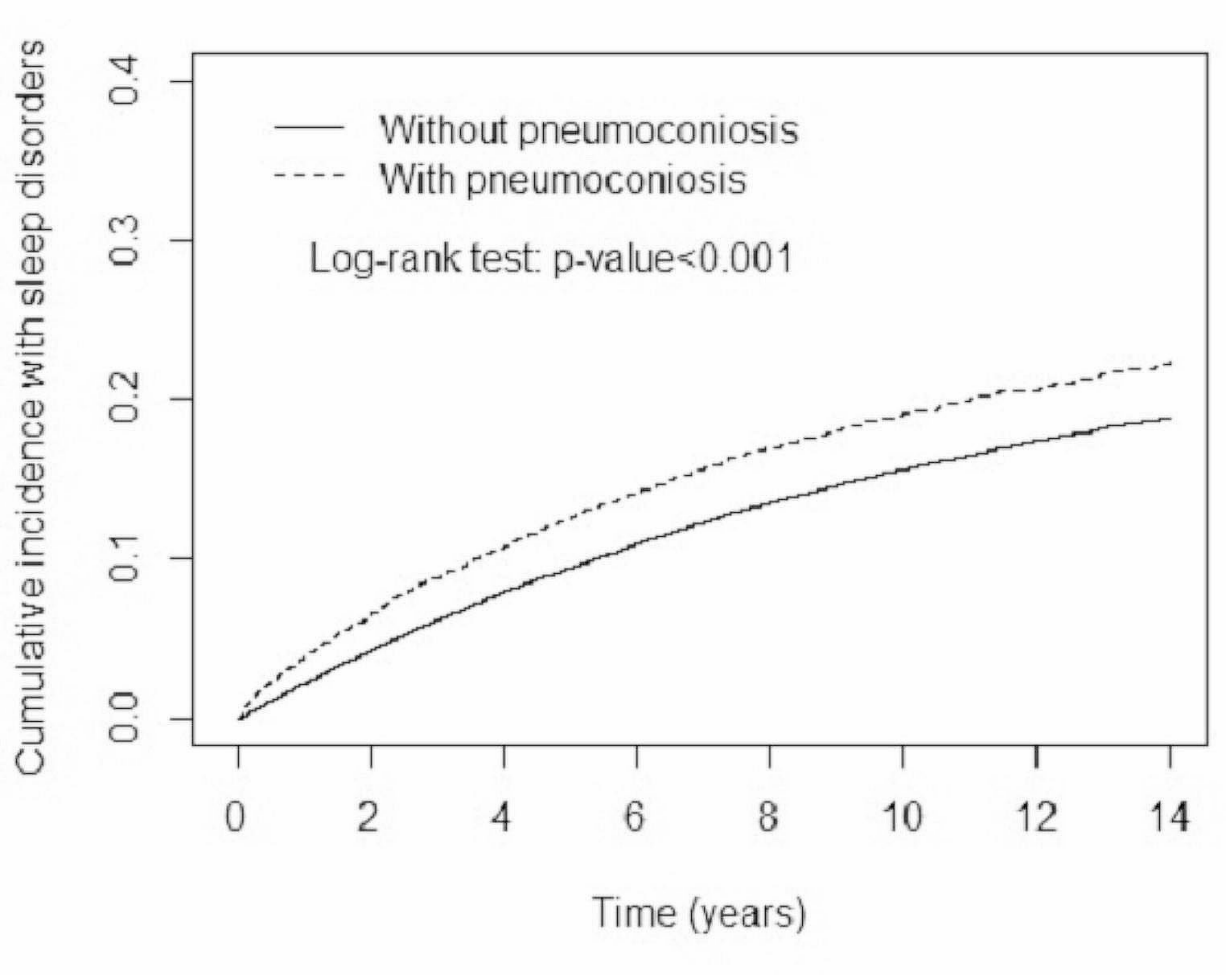
Cumulative incidence of sleep disorders for individuals with and without pneumoconiosis

During the follow-up period, the cumulative incidence of sleep disorders was significantly higher in the pneumoconiosis cohort than in the comparison cohort (*p* < 0.001 in the log-rank test, Fig. 2). The overall incidence of sleep disorders was 1.31-fold higher in the pneumoconiosis cohort than in the comparison cohort (22.8 vs. 16.2 per 1000 person-years, respectively) with an aHR of 1.24 (95% CI 1.17–1.32) after adjusting for age, sex, comorbidity, and medication (Table 2). The aHRs of sleep disorders were 1.26-fold higher in women than in men (95% CI 1.17–1.34). In addition, the risk of sleep disorders was significantly higher in patients with hypertension (aHR 1.31, 95% CI 1.25–1.37), asthma/COPD (aHR 1.55, 95% CI 1.46–1.64), and tuberculosis (aHR 1.34, 95% CI 1.28–1.41).

**Table 2:** Risk factor analyses for sleep disorders among all study individuals

	Event	PY	Rate ^†^	Crude HR (95% CI)	Adjusted HR ^#^ (95% CI)
Pneumoconiosis					
No	7277	448,800	16.2	1.00	1.00
Yes	1480	65,026	22.8	1.31 (1.24 − 1.38) ***	1.24 (1.17 − 1.32) ***
Age					
20 − 49	263	35,717	7.4	1.00	1.00
50 − 64	1904	125,769	15.1	2.02 (1.78 − 2.30) ***	2.01 (1.76 − 2.29) ***
≥ 65	6590	352,339	18.7	2.31 (2.04 − 2.61) ***	2.14 (1.89 − 2.43) ***
Gender					
Men	7812	466,977	16.7	1.00	1.00
Women	945	46,849	20.2	1.23 (1.15 − 1.32) ***	1.26 (1.17 − 1.34) ***
Comorbidity					
Hypertension					
No	3771	277,896	13.6	1.00	1.00
Yes	4986	235,930	21.1	1.46 (1.40 − 1.52) ***	1.31 (1.25 − 1.37) ***
Diabetes mellitus					
No	8560	500,655	17.1	1.00	1.00
Yes	197	13,171	15.0	0.77 (0.67 − 0.89) ***	0.60 (0.52 − 0.69) ***
Hyperlipidemia					
No	6473	401,863	16.1	1.00	1.00
Yes	2284	111,963	20.1	1.20 (1.15 − 1.26) ***	1.02 (0.97 − 1.08)
CVD					
No	7697	467,383	16.5	1.00	1.00
Yes	1060	46,443	22.8	1.26 (1.18 − 1.34) ***	1.03 (0.97 − 1.11)
Asthma/COPD					
No	6918	441,255	15.7	1.00	1.00
Yes	1839	72,571	25.3	1.50 (1.42 − 1.58) ***	1.55 (1.46 − 1.64) ***
CLD					
No	8115	480,082	16.9	1.00	1.00
Yes	642	33,744	19.0	1.03 (0.95 − 1.12)	0.93 (0.86 − 1.01)
CKD					
No	8650	508,845	17.0	1.00	1.00
Yes	107	4981	21.5	1.09 (0.90 − 1.32)	0.89 (0.73 − 1.08)
Tuberculosis					
No	8543	504,672	16.9	1.00	1.00
Yes	214	9154	23.4	1.45 (1.37 − 1.52) ***	1.34 (1.28 − 1.41) ***
Obesity					
No	8740	512,657	17.1	1.00	1.00
Yes	17	1169	14.6	0.83 (0.51 − 1.33)	0.75 (0.47 − 1.21)
Tobacco use disorders					
No	8702	511,060	17.0	1.00	1.00
Yes	55	2766	19.9	1.03 (0.79 − 1.34)	1.00 (0.76 − 1.30)

CI, confidence interval; CKD, chronic kidney disease; CLD, chronic liver disease and cirrhosis; COPD, chronic obstructive pulmonary disease; CVD, cerebrovascular disease; HR, hazard ratio; PY, person-years

^†^ Incidence rate per 1,000 person-years

^#^ Multivariable analysis including age, gender, comorbidity, and medication

*** *p* < 0.001

Table 3 presents data on the incidences and aHRs of sleep disorders in the pneumoconiosis and comparison cohorts, stratified by age, sex, and comorbidities. Among individuals aged 20–49, 50–64, and ≥ 65 years, compared with the comparison cohort, the age-specific aHRs in the pneumoconiosis cohort were 1.64 (95% CI 1.21–2.23), 1.35 (95% CI 1.19–1.54), and 1.19 (95% CI 1.10–1.28), respectively. The sex-specific aHRs were 1.24 (95% CI 1.16–1.32) in men and 1.29 (95% CI 1.08–1.53) in women. For comorbidity, the aHRs were 1.38 (95% CI 1.30–1.47) and 1.23 (95% CI = 1.04–1.46) in those with and without comorbidities, respectively.

**Table 3:** Incidences and hazard ratios of for individuals with and without pneumoconiosis by age, gender, comorbidity

	Pneumoconiosis							
	No			Yes				
	Event	PY	Rate ^†^	Event	PY	Rate ^†^	Crude HR (95% CI)	Adjusted HR ^#^ (95% CI)
Age								
20 − 49	187	30,389	6.2	76	5,329	14.3	2.22 (1.70 − 2.90) ***	1.64 (1.21 − 2.23) **
50 − 64	1534	109,077	14.1	370	16,691	22.2	1.47 (1.31 − 1.65) ***	1.35 (1.19 − 1.54) ***
≥ 65	5556	309,334	18.0	1034	43,006	24.0	1.24 (1.16 − 1.33) ***	1.19 (1.10 − 1.28) ***
Gender								
Women	775	40,442	19.2	170	6407	26.5	1.30 (1.10 − 1.54) **	1.29 (1.08 − 1.53) **
Men	6502	408,358	15.9	1310	58,619	22.4	1.31 (1.23 − 1.39) ***	1.24 (1.16 − 1.32) ***
Comorbidity ^‡^								
No	2010	180,748	11.1	148	11,432	13.0	1.12 (0.95 − 1.33)	1.23 (1.04 − 1.46) *
Yes	5267	268,053	19.7	1332	53,593	24.9	1.20 (1.13 − 1.27) ***	1.38 (1.30 − 1.47) ***

CI, confidence interval; HR, hazard ratio; PY, person-years

^†^ Incidence rate per 1,000 person-years

^#^ Multivariable analysis including age, gender, comorbidity, and medication

^‡^ Individuals with any comorbidity of hypertension, diabetes mellitus, hyperlipidemia, CVD, asthma/COPD, CLD, CKD, tuberculosis, obesity, and tobacco use disorders were classified into the comorbidity group

* *p* < 0.1, ** *p* < 0.01, *** *p* < 0.001

Table 4 presents information on the risk of SA in the pneumoconiosis and comparison cohorts. The aHRs of SA was 1.71 (95% CI 1.31–2.22) in the pneumoconiosis and comparison cohorts, respectively.

**Table 4:** Incidences and hazard ratios of sleep apnea for individuals with and without pneumoconiosis by age, gender, comorbidity

	Pneumoconiosis							
	No			Yes				
	Event	PY	Rate ^†^	Event	PY	Rate ^†^	Crude HR (95% CI)	Adjusted HR ^#^ (95% CI)
Overall	345	448,800	0.77	86	65,026	1.32	2.17 (1.71 − 2.75) ***	1.71 (1.31 − 2.22) ***
Age								
20 − 49	38	30,389	1.25	6	5329	1.13	1.03 (0.44 − 2.44)	1.08 (0.43 − 2.70)
50 − 64	116	109,077	1.06	17	16,691	1.02	1.31 (0.79 − 2.19)	0.94 (0.53 − 1.68)
≥ 65	191	309,334	0.62	63	43,006	1.46	2.95 (2.21 − 3.92) ***	2.49 (1.81 − 3.42) ***
Gender								
Women	13	40,442	0.32	6	6407	0.94	3.18 (1.21 − 8.38) *	2.65 (0.95 − 7.43)
Men	332	408,358	0.81	80	58,619	1.36	2.15 (1.68 − 2.75) ***	1.65 (1.26 − 2.17) ***
Comorbidity ^‡^								
No	90	180,748	0.50	11	11,432	0.96	2.35 (1.26 − 4.40) **	2.33 (1.24 − 4.41) **
Yes	255	268,053	0.95	75	53,593	1.40	1.74 (1.34 − 2.25) ***	1.71 (1.31 − 2.23) ***

CI, confidence interval; HR, hazard ratio; PY, person-years

^†^ Incidence rate per 1,000 person-years

^#^ Multivariable analysis including age, gender, comorbidity, and medication

^‡^ Individuals with any comorbidity of hypertension, diabetes mellitus, hyperlipidemia, CVD, asthma/COPD, CLD, CKD, tuberculosis, obesity, and tobacco use disorders were classified into the comorbidity group

* *p* < 0.1, ** *p* < 0.01, *** *p* < 0.001

## Discussion

As the first of its kind, this retrospective cohort study investigated the association between pneumoconiosis and the development of sleep disorders using nationwide population-based data. The study found that the risk of sleep disorders was 31% higher for people with pneumoconiosis than those without pneumoconiosis. This association was observed across all subgroups analyzed, including those stratified by age, sex, and comorbidities. In addition, patients with pneumoconiosis had a significant higher risk of SA than those without pneumoconiosis. The study findings provide important epidemiological insights into the relationship between pneumoconiosis and sleep disorders on a large scale.

The mechanisms between pneumoconiosis and sleep disorders are unconclusive. Respiratory symptoms, oxygen desaturation, and disruption of the normal sleep architecture may play an essential role. This poor sleep quality may further negatively affect the quality of life, daytime functioning, chronic fatigue, stress, depressive mood, disease progression, cardiovascular comorbidity, and even survival [27, 28].

The association between pneumoconiosis and SA should be more emphasized. SA has a link closer to severe cardiovascular comorbidities, such as CHF, CAD, and CVD. Therefore, cumulative evidence has shown that SA worsens disease progression and mortality in patients with ILD [29, 30]. A recent meta-analysis identified a 61% prevalence of obstructive SA among patients with various forms of ILD, with 26% having moderate-to-severe disease [31]. The mechanism may involve decreased respiratory muscle mass, decreased lung volume increased breathing work, decreased gas exchange, and corticosteroid treatments [32]. Regarding pneumoconiosis and SA sharing comparable cardiovascular risk and may have a synergy effect, the existence of SA in patients with pneumoconiosis must be detected early.

A key study strength is the inclusion of a large pneumoconiosis cohort and a well-matched comparison cohort, with a high rate of follow-up completion. Given the high costs of conducting a prospective cohort study, the use of the Taiwan National Health Insurance database for a retrospective cohort study was an appropriate and cost-effective alternative. In addition, the study accurately reflects real-world scenarios in which pneumoconiosis, sleep disorders, and all comorbidities were diagnosed during medical consultations [33–35].

However, this study has some limitations that should be considered. First, the diagnosis of pneumoconiosis, sleep disorders, and comorbidities relied on the accuracy and competence of clinical physicians using ICD codes. Second, important information such as occupational history, smoking habits, physical activity, and family history were not included in the database, which could have influenced the results. Third, clinical variables such as the pneumoconiosis stage, body weight or body mass index, laboratory data, pulmonary function tests, and imaging results were not available for analysis.

## Conclusion

Patients with pneumoconiosis have a significantly higher risk of developing sleep disorders than those without pneumoconiosis. Moreover, the risk of SA was also significantly higher in patients with pneumoconiosis. Healthcare professionals should pay more attention to sleep quality and disturbance in such patients.

## Electronic Supplementary Material

Below is the link to the electronic supplementary material.

Supplementary Material 1

## Data Availability

All data generated or analyzed during this study are included in this manuscript.

## Abbreviations

aHR: Adjusted hazard ratio
CAD: Coronary artery disease
CHF: Congestive heart failure
cHR: Crude hazard ratio
CI: Confidence interval
CKD: Chronic kidney disease
CLD: Chronic liver disease and cirrhosis
COPD: Chronic obstructive pulmonary disease
CVD: Cerebrovascular disease
ICD: International Classification of Diseases
ILD: Interstitial lung diseases
NHI: National Health Insurance
SA: Sleep apnea
