# Supplementary material for: Risk of sleep disorders in patients with pneumoconiosis: a retrospective cohort study
Source: J Epidemiol Glob Health. 2024 Apr 4;14(3):860–8. doi: 10.1007/s44197-024-00225-5 (PMC11442832; doi:10.1007/s44197-024-00225-5)
Supplement: Supplementary file 1 — Supplementary Material 1 [file 44197_2024_225_MOESM1_ESM.pdf]

職業性矽肺症及煤礦工作塵肺症認定參考指引

勞動部職業安全衛生署

中華民國110年6月

【本參考指引由勞動部職業安全衛生署委託**蔣得明、白宗平、羅錦泉醫師**主筆修定】

一、導論

塵肺症係指粉塵沈積在肺內，所造成的非腫瘤性組織反應[1-3]；因職業上原因吸入礦物等粉塵，粉塵沈著而引起之肺部疾病則稱為職業性塵肺症[4]。

依據現行我國勞工作業場所容許暴露標準，將空氣中粉塵分為四種：第一、二種為與游離二氧化矽相關的礦物性粉塵、第三種為石綿纖維、第四種為厭惡性粉塵。粉塵是否容易進入人體與其粒徑大小有關，一般來說，粒徑100 μm以下稱為可吸入性粉塵，10 μm以下稱胸腔性粉塵，4 μm以下稱可呼吸性粉塵，其中可呼吸性粉塵因粒徑最小可深入並沈積在肺部，對健康危害性最大。目前因粉塵作業引起的職業病指引如表一。

表一、職安署公告因粉塵作業引起的職業病認定參考指引

**目標疾病**	**職業暴露**	**職業病種類表項目**	**職業病認定參考指引**	**修訂時間**
塵肺症		第 8 類<br>第 1 項	**職業性矽肺症及煤礦工人塵肺症認定參考指引**	本指引
矽肺症合併肺癌	結晶型游離二氧化矽粉塵	增列<br>第 5.20 項	**職業暴露結晶型游離二氧化矽粉塵引起之矽肺症合併肺癌認定參考指引**	2018
肺癌	含石綿的滑石	增列<br>第 5.1 項	**包括含石綿的滑石-引起之職業性癌症診斷認定參考指引-3 肺癌**	2009
肺癌	石綿外物質	-	**石綿外-引起之職業性癌症診斷認定參考指引-3 肺癌-98 年訂定一版**	2009
肺癌-鼻竇癌-鼻癌	無機鎳及其化合物	增列<br>第 5.14 項	**無機鎳及其化合物引起的職業性肺癌-鼻竇癌-鼻癌認定參考指引**	2015
職業性肺疾病	鋁	增列<br>第 7.1 項	**鋁引起的職業性肺疾病認定參考指引**	2016
職業性肺病	硬金屬<br>如鈷-鎢-鉬-鈦-鉻及其他	增列<br>第 7.2 項	**硬金屬肺病-如鈷-鎢-鉬-鈦-鉻及其他硬金屬引起的職業性肺病認定參考指引**	2015
石綿肺症	石綿	增列<br>第 7.4 項	**職業暴露石綿引起之石綿肺症認定參考指引**	2018
慢性阻塞性肺病	地下礦工	增列<br>第 7.6 項	**地下礦工的慢性阻塞性肺病認定參考指引**	2015
肺部疾病	有機粉塵	增列<br>第 7.6 項	**職業暴露有機粉塵引起之肺部疾病認定參考指引**	2018

台灣常見的塵肺症有矽肺症(silicosis)和煤礦工人塵肺症(coal workers' pneumoconiosis)[5-16]；至於石綿肺症(asbestosis)，在台灣迄今只有數例報告[17-19] ；近十年來中國的塵肺症原因前三名則為矽肺症、煤礦工人塵肺症以及電焊工人塵肺症(welder's pneumoconiosis)[20]。

此次修訂將原本的職業性矽肺症及煤礦工人塵肺症認定參考指引，調整為職業性塵肺症認定標準，主要探討矽肺症、煤塵肺疾病及電焊工人塵肺症，其中煤礦暴露引起的慢性阻塞型肺疾病請參考" 地下礦工的慢性阻塞性肺病認定參考指引"，其他粉塵作業引起的肺部疾病請參考表一。

(一)矽肺症

矽肺症為最常導致失能且最為人知的塵肺症，主要由二氧化矽沉積在肺中導致。而二氧化矽依型態分為結晶型游離二氧化矽(crystalline free silica)，如石英(quartz)、方矽石(cristobalite)以及鱗矽石(tridymite)；隱晶型(cryptocrystalline)如石髓(chalcedony)；非結晶型態(amorphous)如蛋白石(opal)、矽藻土(diatomaceous earth)。其中結晶型游離二氧化矽在自然界中分佈甚廣，為岩石砂土的主要成分，許多工業的作業過程中常會產生矽石的微粒，如表二所描述其化學特性穩定，溶解度低，進入肺部便不易排出，沈積於肺部的結晶型二氧化矽會造成肺部組織纖維化，產生不可逆之病變稱為矽肺症；初期在肺內形成細小而各自分離的纖維性小結，這些矽肺小結直徑約1-10公厘，分佈於血管周圍的淋巴管，胸膜下及胸腔內的淋巴結中。肺的上部和肺門淋巴結所受的侵犯要比肺的底部嚴重。矽肺小結會漸漸融合而成為直徑大於1公分的進行性大塊纖維化(progressive massive fibrosis, PMF)[21-24]，歐盟已將肺部電腦斷層放入診斷基準當中，表示其比胸部X光更具敏感性[25]。而國際癌症研究機構(The International Agency for Research on Cancer; IARC)經多年流行病學研究之資料證實結晶型游離二氧化矽之危害，在1997年將其由致癌物質分類Group 2A改為Group 1，即確定人類致癌物質[26]。

結晶型游離二氧化矽對人體的危害，以矽肺症最廣為人知，除此之外，還有慢性阻塞型肺疾病、肺癌、自體免疫疾病以及腎病變[25]。矽肺症可分為慢性矽肺症(chronic silicosis)，加速型矽肺症(accelerated silicosis)以及急性矽肺症(acute silicosis)[27]。急性矽肺症是進行甚速的疾病，暴露於高濃度的可呼吸性粉塵(矽)中數週至4-5年後發病，此類型的矽肺症多來自於噴砂業[27]，病人往往在出現進行性呼吸困難、咳嗽、體重減輕、疲倦等症狀後一年內死亡[28]。加速型矽肺症在暴露後5-10年內發病，和慢性矽肺症的臨床表現相似，但進展速度較快。慢性矽肺症最為常見，通常在暴露矽塵約10年後發生，進行緩慢。不管是急性或慢性矽肺症，即使停止矽塵的暴露，肺內的纖維化過程仍會繼續進行。矽肺症常合併肺結核[29-30]，也常進行至呼吸衰竭或併發肺心症。

(二)煤塵肺疾病(coal mine dust lung disease, CMDLD)

煤礦工人塵肺症主要發生於煤礦工人，因吸入煤塵而致。細支氣管周圍由於煤塵及噬入煤塵的吞噬細胞聚積而形成煤塵斑。煤塵斑逐漸增大並雜有不同程度的纖維化，乃形成煤塵結節，常合併局部性中央小葉型肺氣腫，煤塵結節繼續增大融合，最後發展成進行性大塊纖維化[31]。其嚴重程度和肺內沈積的粉塵總量密切相關[32-36]。此類病人發生支氣管炎及肺氣腫的情形和程度，也和煤塵暴露的程度有關[37-41]。

煤塵肺疾病是吸入煤塵導致，除了上述典型的煤礦工人塵肺症以及進行性大塊纖維化外，還包括同時暴露二氧化矽粉塵的混合性塵肺症、慢性氣管炎、肺氣腫及粉塵相關的廣泛性纖維化(dust-related diffuse fibrosis)等，肺功能檢查為以侷限性變化為主；同時煤塵暴露也會使肺功能產生阻塞性變化，產生類似慢性肺阻塞性疾病的表現，在曾經抽菸者身上尤其明顯[42]。

(三)電焊工人塵肺症

電焊工人塵肺症是由吸入氧化鐵燻煙、粉塵(化學特性如表二所描述)或其他電焊燻煙造成的，胸部X光明顯異常的個案往往沒有異常的肺功能或呼吸道症狀，因此最初被認為是良性的，此類疾病也被稱為肺鐵質沉積症(pulmonary siderosis)，然而後續的研究則發現仍有個案產生肺部纖維化甚至造成肺功能異常，目前有的證據大多為個案報告(case reports)及系列個案報告(case series)且文獻較舊，且在歐盟的指引中將本疾病描述為良性的塵肺症。個案的臨床表現為呼吸困難、持續咳嗽或異常的胸部X光且同時有長期暴露於電焊燻煙中的暴露史，很少產生杵狀指(clubbing finger)且偶而會有囉音(crackles)、鐵過量(iron overload)、肺因性心臟病(cor pulmonale)或融合成大塊病灶(conglomeration)的狀況；在有肺纖維化證據的個案中，肺功能檢查結果可能為阻塞型變化，也有以限制型、混合型甚至是正常的肺功能表現，吸菸與電焊對於肺部有協同作用(synergic)的影響，使得肺功能進一步下降；肺部電腦斷層的特徵為毛玻璃變化(ground glass changes)；肺部組織檢查搭配礦物調查顯示，肺部纖維化區域沒有結晶型二氧化矽或是石綿的成分，但與電焊燻煙顆粒有高度關聯性 [43, 44]。在鐵過量的個案中，一些個案的血中儲鐵蛋白(ferritin)會上升，可作為生物偵測的參考指標 [45]。

表二、我國常見產生塵肺症物質的性質[46]

結晶型游離二氧化矽			
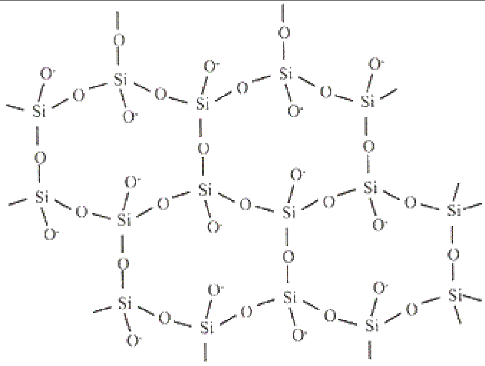			
**IUPAC名稱**	Crystalline silica		
**CAS編號**	14808-60-7	**密度**	2.66 g/cm^3
**分子式**	SiO_2	**水中溶解度**	不可溶解
**分子量**	60.1 g/mol	**蒸氣壓**	0 mmHg(886°C)
**1大氣壓常溫狀態：**		**外觀**	無色
**熔點**	1710°C	**味道**	無味
		**IARC**	Group 1

氧化鐵			
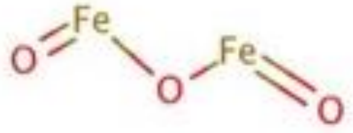			
**IUPAC名稱**	Iron(III) oxide		
**CAS編號**	1309-37-1	**密度**	5.24 g/cm^3
**分子式**	Fe_2O_3	**水中溶解度**	不可溶解
**分子量**	159.69 g/mol	**蒸氣壓**	-
**1大氣壓常溫狀態：**		**外觀**	紅棕色
**熔點**	1566°C	**味道**	無味
		**IARC**	N/A

二、具潛在暴露之職業

(一)矽肺症

1.採礦(mining)、隧道工程(tunneling) [5, 6, 7, 8, 9, 10, 25, 47, 54, 73]

2.採石業(quarrying of sand, sandstone, slate) [25, 47, 55]

3.石匠(mansory) 、雕刻師(sculpture) [25,47]

4.鑄造工(foundry work) [25, 47, 48, 75]

5.鼓風爐(blast furnaces)修補相關工程 [25,47,75]

6.噴砂業(sand blasting) [25,47]

7.砂石研磨(sandstone milling and grinding) [25,47, 48]

8.製造或使用含碳化矽(carborundum)的研磨劑(abrasives) [25,47]

9.玻璃製造業(glassmaking) [25,47, 48]

10.玻璃搪瓷(vitreous enamelling) [25,47, 48]

11.陶瓷製造業(ceramic manufacture) [13, 25, 47, 48, 76, 77]

12.使用結晶型二氧化矽作為填充物的顏料(paint)、橡膠(rubber)、木工(woodworking) [25,47]

13.石材製品製造業 [48, 74, 78]

表三、目前台灣使用含結晶型游離二氧化矽的礦物原料之主要行業[48]

原料	使用行業	結晶型含量%
石英岩	矽鐵工廠、耐火磚業	90-99
石英砂（矽砂）	矽砂工廠、鑄造業、玻璃業、砂紙製造業、耐火磚業	85-99
黏土	耐火磚業、瓷磚業、衛浴瓷器、日用瓷器、藝術陶瓷、蘭草加工廠、窯爐製造業	0-60
花崗岩	建築業（石材）	25-30
瑪瑙、水晶	寶石琢磨加工業	>98

(二)煤塵肺疾病

1.煤礦採礦、修整、運送[13, 15, 25, 47, 56, 57, 58, 61, 62, 63, 71, 72]

2.碳黑(carbon black)生產與使用、碳電極製造[25,47]

3.石墨採礦[25,47]

(三)電焊工人塵肺症

1.鋼鐵軋製或研磨(iron and steel rolling or grinding) [25]

2.鑄件加工修整 (fettling) [25]

3.電焊(welding)，如電弧焊(electric arc)、氧乙炔焊(oxyacetylene welding)。[25, 43, 44, 45, 47, 64, 65, 66, 67, 68]

4.其他可能造成肺鐵沈著症之職業：用氧化鐵粉末拋光金屬、玻璃或石頭；鍋爐水垢(boiler scaling)；採挖鐵礦(mining or crushing iron ore)；開挖、研磨金剛砂(mining or milling of emery)；製造磁帶(manufacture of magnetic tapes)；製造顏料(manufacture of pigments)。[25]

三、醫學評估與鑑別診斷

(一)臨床症狀

單純型塵肺症常無症狀，複雜型塵肺症常有咳嗽、呼吸困難、胸痛、發紺、喘鳴、囉音等。

(二)胸部X光

慢性矽肺症或加速型矽肺症的表現為雙側肺部多顆圓型陰影，大多出現在肺部的上三分之一處，且隨著疾病進展可出現圓形陰影聚集成團；急性矽肺症則以雙側肺泡浸潤表現為主。

1.依照國際勞工聯盟(International Labour Organization, ILO)塵肺症X光國際分類法記載胸部X光，記載項目如下[49]。

(1)小陰影大小(最大直徑一公分以下者) ：

p：圓形陰影其直徑 <1.5mm。

q：圓形陰影其直徑 1.5-3 mm。

r：圓形陰影其直徑為 3-10 mm。

s：細的不規則或線狀陰影。

t：較粗的不規則陰影。

u：粗的不規則陰影。

(2)密度分布

1:稀疏：正常肺紋理(lung marking)明顯可見

2:密集：圓形陰影之正常肺紋理仍然明顯可見；不規則陰影之正常肺紋理部分模糊不清。

3:極密：圓形陰影之正常肺紋理部分或全部模糊不清；不規則陰影之正常肺紋理全部模糊不清。

(3)大陰影(最大直徑一公分以上者)

A：大陰影總和直徑 <5cm。

B：介於A與C兩者間之大陰影。

C：大陰影總面積超過右側三分之一上肺野者。

2.塵肺症X光照像分型基準（勞工保險塵肺症審定準則，民84年）

(1)第一型：在兩肺野有因塵肺發生之少數粒狀影或不規則陰影，且無大陰影者。

(2)第二型：在兩肺野有因塵肺發生之多數粒狀影或不規則陰影，且無大陰影者。

(3)第三型：在兩肺野有因塵肺發生之極多粒狀影或不規則陰影，且無大陰影者。

(4)第四型：有大陰影者

(三)肺功能檢查 [21-22，33-34，50-52]

嚴重的塵肺症可能有下列的變化

1.用力呼氣肺活量(FVC)減低

2.第一秒用力呼氣量(FEV_1)減低

3.一氧化碳瀰散量(DLCO)減低

4.最大呼吸量(MVV)降低

5.動脈血氧飽和度在運動時減低

(四)鑑別診斷

1.Pneumoconiosis due to dust containing silica (含矽粉塵所致塵肺症)之國際疾病分類診斷碼ICD10為J62.8。

2.Coal worker's pneumoconiosis(煤礦工人塵肺症)之國際疾病分類診斷碼ICD10為J60。

3.Siderosis (鐵末沈積症)之國際疾病分類診斷碼ICD10為J63.4。

4.Pneumoconiosis due to other specified inorganic dusts (其他特定無機性塵埃所致之塵肺症)之國際疾病分類診斷碼ICD10為J63.6。

5.Unspecified pneumoconiosis (塵肺症) 之國際疾病分類診斷碼ICD10為J64。

6.肺結核。

7.癌症。

8.纖維化性肺泡炎(fibrosing alveolitis) 。

9.類肉瘤症(sarcoidosis)。

四、流行病學證據

已有相當多的流行病學證據證明了結晶型游離二氧化矽及煤塵與矽肺症及煤礦工人塵肺症之間的關係。相較之下，非結晶型的二氧化矽目前被認為對人體無害，但若是當中含有結晶型二氧化矽仍有可能產生肺部纖維化，如鍛燒過的矽藻土(calcined diatomaceous earth)等[27, 53] 。暴露到其他的商用型矽酸鹽化合物引起的塵肺症也較為罕見，臨床表現相對良性，可能只有輕微的纖維化，在歐盟的指引[25] 中提及這些的塵肺症案例可能是被石英或是石綿纖維污染所致；上述常見的商用型矽酸鹽化合物包含頁矽酸鹽類(phyllosilicate)的黏土礦物(clay mineral)如高嶺土(kaolin)、漂白土(fuller's earth)以及非黏土的頁矽酸鹽類(non-clay phyllosilicate)如滑石(talc)及雲母(mica)。

Dreessen觀察暴露於游離矽含量為20-40%的727名金屬礦工，其中暴露在矽塵濃度為10-23 mppcf (mi1lion particles per cubic foot)達六年以上者，得矽肺症者有25%；而在矽塵濃度低於18 mppcf下暴露未超過

10年者，沒有一個勞工發生矽肺症。矽肺症者肺纖維化的嚴重程度隨受雇年數的增加而加重[54]；美國職業安全衛生署(Occupational Safety and Health Administration, OSHA)採用轉換係數(conversion factor)為1 mppcf = 0.1 mg/m^{3}可呼吸性粉塵。

Theriault 對49個花崗岩房的784位勞工調查，發現暴露於花崗岩粉塵和石英勞工的用力吐氣肺活量、第一秒用力呼氣量及全肺容量(total lung capacity) 降低程度與暴露粉塵時間正相關，其中223位的胸部X光有矽肺症的變化、而X光異常者其矽塵暴露量較X光正常者高出2.3倍[55]。

Jacobsen發現煤礦工人塵肺症胸部X光的嚴重程度和所暴露的可呼吸性煤塵量正相關[56]；Walton觀察到煤礦工人塵肺症的致病率(attack rate)和暴露期間的可呼吸性煤塵量正相關[57]，Rossiter發現肺內沉積的煤塵量及其他礦塵量也和暴露期間的可呼吸性煤塵量有關[58-60]。

Almberg及Kurth等人在美國國家職業安全衛生研究所(National Institute for Occupational Safety and Health, NIOSH)的資助下於2018-2020年分析了聯邦黑肺症計畫(The Federal Black Lung Program, FBLP)的資料[61-63]，在1970-2016年期間總共有314,176位礦工申請補助，其中有4,679位有進行性大塊纖維化(PMF)，有PMF者平均年齡61.6歲、平均工作年資23年，比non-PMF者年資(平均18.9年)較長；在1999-2016年期間，黑肺症計畫(FBLP)患者的呼吸及心臟相關疾病盛行率比其他患者高、住院率也較高，當中最常見的共病為阻塞性肺病(87.6%，相較其他患者：11.6%)、高血壓(81.6%，其他患者：56.9%)及缺血性心臟病(80.0%，其他患者：27.0%)，前三大死因為阻塞性肺病(10.1%)、粥狀動脈硬化心臟病(atherosclerotic heart disease，9.3%)及煤礦工人塵肺症(9.2%)。

Doig和McLaughlin 於1936年首次在電焊工人的胸部X光中發現電焊工人塵肺症，這些個案無症狀，也沒有接觸過二氧化矽或煤礦，因此

被列為一種新的塵肺病類型；後續追蹤9年的2名個案，其中一名離職的電焊工胸部X光完全恢復了正常，另一名工人後續顯著減少接觸電焊燻煙，其肺部陰影也部分減少[64-66]。

Morgan和Kerr於1963年研究了7名X光異常的電焊工人，其中3名有輕微的呼吸道症狀，組織學檢查顯示肺泡有大量的鐵沈積同時有輕度的組織間纖維化；其中一名有顯著肺部纖維化的工人被歸因為過去的肺結核[67]。

Funahashi於1988年研究了10名有症狀的電焊工人，其中有5名出現中度肺纖維化，肺功能檢查發現7名焊工有侷限性肺病，2人出現輕到中度阻塞性肺病，3人肺泡擴散能力(diffusion capacity)降低；此研究中肺部有大量鐵沈積，且發現含鐵顆粒出現在纖維化的肺泡間隔(alveolar septa)，但矽的含量跟對照組無差別，因此認為肺部的間質性纖維化可能與鐵有關[68]。

近期的縱貫式研究的統合分析顯示焊接燻煙的暴露在肺功能上沒有顯著的變化(FEV_1及FVC)，然而橫斷性研究顯示有些電焊工有阻塞性肺病的證據，有些則以侷限性肺病表現；次群體分析顯示，沒有使用局部通風者肺功能(FEV_1及FVC)也有顯著的下降[43]。

Cosgrove於2015年進行了一次文獻回顧，涵蓋了除了鋁電焊造成的肺部疾病及電焊燻煙造成的急性肺炎(acute pneumonitis)外的鋼鐵電焊肺疾病相關的文獻，包含了12個系列個案報告與34個個案報告，並於2014年三次的專家會議討論後發表，當中提到Buerke等人發表的系列個案報告中，病患以呼吸困難、持續咳嗽或異常的胸部X光且同時有長期暴露於電焊燻煙中的暴露史。個案很少產生杵狀指且偶而會有囉音、鐵過量、肺因性心臟病或融合成大塊病灶的狀況；停止電焊燻煙以及抽菸的暴露很可能可以避免症狀惡化，但仍須持續追蹤[43,44]。

在有肺纖維化證據的個案中，肺功能檢查可能為阻塞型變化，也有以限制型、混合型甚至是正常的肺功能表現，吸菸與電焊對於肺部有協同作用的影響，會使得肺功能進一步下降；肺部電腦斷層提供的

訊息比肺功能檢查多，特徵為毛玻璃變化，某些肺纖維化個案當中有肺氣腫(emphysema)產生，但這些個案也常有抽菸史[43]。

活體肺切片並進行纖維化區域的礦物分析可提供近一步資訊，Müller和Verhoff將因為暴露於電焊燻煙產生肺部纖維化的病理發現分成輕度、中度及嚴重程度。輕度個案中肺泡及間質中有巨噬細胞，但在粉塵沈積處無纖維化；中度個案有巨噬細胞數目增加，並且在間質粉塵沉積處有顯著的纖維化；嚴重個案的特徵為大量的沉積物、局部增加的慢性纖維化以及沉積物與纖維化的高度關聯性。另外若有下述兩個特徵，則較可能因職業暴露導致：1.離開暴露後，個案的臨床症狀不再進展、2.肺部組織檢查搭配礦物調查顯示，肺部纖維化區域沒有結晶型二氧化矽或是石綿的成分，而與電焊燻煙顆粒有高度關聯性 [43]。

此外有文獻指出某些個案有鐵過量的特徵出現，血中儲鐵蛋白會上升，可作為生物偵測的參考指標；在這些個案中儲鐵蛋白的血中濃度上升至1000-2000 ug/L之間，且血中鐵及TIBC正常，血清運電蛋白飽和度(transferrin saturation)略為上升，而經由放血治療(venesection)16至18個月後，這些個案的儲鐵蛋白回到正常值[45]。

[台灣流行病學資料]

依據臺灣本土的流行病學證據，塵肺症在台灣是最早被報告的肺部職業病[69]。楊思標醫師在1952年報告6例塵肺症的病例[5]，其中兩例是礦工矽肺症、兩例是鐵工廠工人塵肺症，一例是燒煤火伕的炭肺症，另一例是雨傘工人得塵肺症的病例。接著楊醫師於1953年瑞芳金瓜石金銅礦山，進行礦工坑內工作者矽肺症的調查[6, 70]，受檢之261名礦工中，92人(35.2%)有矽肺症；金瓜石礦山的主要礦石為金礦、硫砷銅礦、黃鐵礦等、礦石內硅酸含有度為70-80%，這些礦工的工作性質包括風鑽工、炮工、扒土工、架攘工等；坑內溫度非常高、工作時是半裸體、未帶口罩；鑿岩機是乾式，無使用防止塵埃發生的特別裝置，工作期間大多在坑內吃中飯。就平均工作年資而言，無矽肺症者6.8年、矽肺症第一期者12.2年、第二期者14.5年、第三期者18.1年；測驗坑

內礦工的胸圍差、肺活量、肺活量呼出時間，呼吸停止時間、以及觀察運動後有無呼吸短促等項目，結果顯示矽肺症患者的肺功能低下。李宣果教授於1954年調查金瓜石金銅礦務局風鑽工罹患矽肺症情形[7]，粉塵中二氧化矽含量約佔80%，受檢139人中罹患矽肺症者57人(41%)，李教授建議改用濕式風鑽機和配戴防塵口罩。經過改善後，李教授於1958年再度調查風鑽工[8]，147位受檢者中矽肺症者11人(7.5%)，罹病人數有明顯下降。柯源卿醫師追蹤271位金瓜石金礦工人10年(1953年至1963年)[9-10]，發現271位礦工已有38人失能、93人(34.3%)死亡；死因中，矽肺症及矽肺症合併肺結核共71人。1956年台灣省防癆局篩檢瑞芳某煤礦廠2,736位礦工[15]，其中64人(2.3%)罹患塵肺症，其中1,748位礦內工作者有55人(3.2%)罹患塵肺症。1966年再度對瑞芳鎮居民篩檢，發現12,195人中罹患塵肺症者有798人(6.5%)。

朱永釗醫師於1969年和1971年兩次調查北部煤礦工人各7,352人和6,047人，結果發現罹患塵肺症者僅1.41%和1.82%。1972年台北市衛生局委託防癆協會第一結核病防治所調查台北市廠礦業塵肺症罹患情形[13]，結果發現煤礦業、窯業和陶瓷業的罹患率分別為2.1%、4.9%和4.0%。1981年朱永釗醫師再對台北市上述三種職業進行調查[15]，發現煤礦業、窯業的塵肺症罹患率分別為14.6%和3.7%，而陶瓷業403位受檢者無罹患塵肺症者。就工齡來看，塵肺症患者服務年資在4年以下者僅有1人，大多數服務年資在15年以上。就工種來看，塵肺症患病率以掘進工與採煤工最高20.3%、其次是支柱工與改修工 10.3%。塵肺症的嚴重程度以第一型最多(55.9%)，二、三、四型分別佔26.5%、11.8%和5.9%。1978年起台灣省衛生處辦理煤礦工人塵肺症的防治[71]，發現歷年來盛行率約在10%左右，到1988年提升至24%。

根據臺灣省礦務局的統計[70]，臺灣地區的煤礦坑口由 1952 年的240個增加到 1966 年的 394 個而達高峰後，急速下降至 1991 年只剩 28 個。員工人數由 1952 年的 40,042 人增至 1967 年的 65,054 人而達高峰後，急速下降至 1991年只有 2,449 人。綜上述資料而言，

1954年，礦工塵肺症盛行率高達35~41%，後因工作方法的改變，1956年起盛行率下降至6~8%，至1978年以後，由於工作年資的增長，塵肺症盛行率升高至10%，到1988年更增加至24%。

楊錫欽等人於2000年收集了 206 位退休煤礦工塵肺症患者資料進行分析[72]：患者原來在基隆、萬里、瑞芳、平溪、三貂嶺、樹林、三峽、頭份等地的煤礦工作，其中男性 182 位（88.3%）、女性 24 位（11.7%），平均年齡67.5 歲，平均工作年資 33.7 年，平均離職年數 14.5 年。以工種來看，坑內 162 人、坑外 44 人；坑內的工種依次是風鑽工 65 人(31.6%)、採礦工 34 人(16.5%)、支柱工 22 人(10.7%)；坑外則大多數是煤塊篩選工 36 人（17.5%），其餘 8 人（3.9%）是維修、雜役工。煤礦工塵肺症會隨著年齡與工作年資的增加而惡化，其 X 光表徵顯示進行性重度肺纖維化或進行性大塊纖維化者佔 21.8%（45 位），而一般在職礦工則低於 10%。

我國行政院勞委會勞工安全衛生研究所於1999年發布”台灣地區塵肺症勞工世代追蹤暨健檢分析研究“中指出[73]，塵肺症勞工有60%為20歲前即開始從事礦業，平均工作年數為36.7年，從開始工作後到診斷出塵肺症大約經歷38年，自覺不適症狀以咳嗽(83%)、呼吸困難(70%)及胸痛(63%)為主。在簡單回歸模式下，平均每增加一年礦業工作年資，減少0.19公升之FEV1及0.01公升之FVC。另294名塵肺症礦工進行胸部X光並以勞保四級分類，結果顯示12名(4%)為第零級、136名(46.3%)為第一級、100名(34%)為第四級，在複回歸模式下胸部X光嚴重程度與肺功能(FEV1、FVC)明顯呈現負相關。

我國勞動部勞動及職業安全衛生研究所於2013年發布“石材製品製造業二氧化矽粉塵暴露危害調查研究”中指出[74]，台灣的石材產業開始於1960年，以生產工藝品為主，到了1970年初，台灣的房屋建築產業開始發展，石材與石板的需求增加，也使得石材產業蓬勃發展。1985年，國內的建築開始流行用色彩鮮豔亮麗的花崗石來裝飾外牆，1986年，進口石材原石關稅降為零，促成了花崗石材的大量使用。近年來大

理石礦開採有總量限制，因此石材改由以進口為主。綜合上述原因，台灣石材產業所使用的原石由大理石逐漸取代成為進口花崗石。石材製品製造業的作業環境因大量使用花崗石，石作工人容易吸入結晶型游離二氧化矽，若石作工人長期暴露於其粉塵下，易導致矽肺症的產生。據行政院勞工委員會之調查資料顯示，以本省各地採回之礦石樣品分析結果，結晶型游離二氧化矽(Crystalline Free Silica）幾乎皆為石英，而一般工業用的花崗岩含石英約25～40%。該研究選擇10 家石材場做現場積塵採樣，於其中3家做現場個人與環境採樣。二氧化矽分析結果顯示，所有粉塵皆含比例不等之二氧化矽，現場環境採樣之平均二氧化矽為8.76%，積塵則高達19.5%。故建議石材製品製造業之粉塵危害，應至少以第二種粉塵標準檢視其作業環境，以保障勞工健康。

林洛秀等人於2010年發表文章，針對鋼鐵廠之煉鐵、煉鋼製程勞工與修爐員工進行探討，整個研究族群共61人；暴露組36人，對照組25人，分析游離二氧化矽暴露與肺功能的相關性，結果發現石英暴露量愈高者百分之二十五的用力呼氣流速(forced expiratory flow 25%)與最大呼氣流速(maximal expiratory flow rate)之檢測值愈低[75]。

我國勞動部勞動及職業安全衛生研究於2014年發布”陶瓷製造業勞工暴露調查研究”中指出，衛生陶瓷工人(sanitary ceramic)為確實將產品坏體內外完全修整和施釉，需以人工方式修整坏體，坏體乾燥後也需以人工澆淋或噴霧方式為坏體上釉，因此相較於其他陶瓷工人，勞工暴露粉塵的機率也較高[76]。曹又中醫師與羅錦泉醫師於2016年發表的文章，以回溯性的方式調查221位診斷為矽肺症的患者，比較衛生陶瓷工人以及其他陶瓷工人後發現，在多變項分析中，兩組的肺功能並無明顯差異；但衛生陶瓷工的FVC以及FEV_1低於80%的風險勝算比(odds ratio)較高[77]。

索任醫師及郭育良醫師於2019年描述一名30歲男性急性惡化的矽肺症個案[78]，主要症狀為乾咳1年多，呼吸喘及體重下降，胸部X光及電腦斷層檢查發現兩下肺廣泛毛玻璃樣變化和兩上肺纖維化，痰和肺

切片皆沒有培養出結核菌，病理學檢查顯示變化符合矽肺症合併肺纖維化及蛋白質沉著症的診斷，且具有侷限性肺功能障礙，後續接受肺臟移植，最後因多重器官衰竭宣告死亡；該勞工從事客製化流理台製作，石英石切割研磨工作年資8年，其中前7年切割石板採用乾式作業，後才引進濕式作業，而修磨石板一直採用乾式作業，工作時粉塵密佈，並於工作前6年僅使用一般外科口罩為個人防護具。結晶型游離二氧化矽含量越高，吸入矽塵量越大，進行速度會較快，若在短時間內高強度暴露於高濃度結晶型游離二氧化矽含量高的可呼吸性矽塵，可發生急性惡化的矽肺症。病理上除了看到密布的矽結節等纖維化之外，也會看到肺泡充滿PAS陽性蛋白質液體，外觀很像肺泡蛋白質症，稱之為矽蛋白質症(Silicoproteinosis)。

五、暴露證據收集方法

(一)作業經歷調查:

詢問勞工過去和目前的職業史，內容包括工作起始年月、部門、職稱、具體作業情形、一般作業環境、整體或局部通風設施、個人防護具的使用等。

(二)作業現場的採樣分析建議方法:

採樣方法可參考勞動部勞動及職業安全衛生研究所標準分析參考方法：4004 總粉塵之結晶型游離二氧化矽、4003 可呼吸性結晶型游離二氧化矽、3007 氧化鐵、煤塵可參考4001可呼吸性粉塵及4002總粉塵中第四種粉塵之方法。

(三)容許濃度標準[46]：

物質		我國勞工作業場所容許暴露標準 mg/m³ (八小時日時量平均容許濃度)	OSHA PEL-TWA mg/m³	NIOSH REL-TWA mg/m³	ACGIH 2019 TLV-TWA mg/m³
結晶型二氧化矽	可呼吸性粉塵	第一種粉塵: 10/(%SiO_2+2)<br>第二種粉塵: 1	石英: 10/(%SiO_2+2)<br><br>方矽石及鱗矽石:以上述計算值之 1/2	石英、方矽石、鱗矽石：0.05	石英、方矽石：0.025
	總粉塵	第一種粉塵: 30/(%SiO_2+2)<br>第二種粉塵: 4	石英: 30/(%SiO_2+2)<br><br>方矽石及鱗矽石:以上述計算值之 1/2	-	-
煤塵	可呼吸性粉塵	第四種粉塵：5	含游離二氧化矽 5%以上之礦物性粉塵：10/(%SiO_2+2)<br><br>含游離二氧化矽未滿 5%之礦物性粉塵：2.4	1	無煙煤:0.4<br>瀝青或褐煤: 0.9
總粉塵		第四種粉塵：10	-	-	-
氧化鐵		燻煙：10	燻煙：10	粉塵及燻煙：5	可呼吸性粉塵：5

備註：第一種粉塵：含游離二氧化矽 10%以上之礦物性粉塵
第二種粉塵：含游離二氧化矽未滿 10%之礦物性粉塵
第四種粉塵：厭惡性粉塵
ACGIH：美國政府工業衛生師協會(American Conference of Governmental Industrial Hygienists)
NIOSH：美國國家職業安全衛生研究所(National Institute for Occupational Safety and Health)

六、結論

(一)主要基準

1.肺部組織因吸入礦物性粉塵或燻煙之纖維化，胸部X光依國際勞工聯盟分類第一型以上。

2.參考歐盟2009年職業病診斷指引[25]：

(1)結晶型二氧化矽暴露相關塵肺症：

A.慢性矽肺症或加速型矽肺症

(A)症狀：症狀可能極輕微、在較為嚴重的情況下可能出現咳嗽以及喘的情形。

(B)胸部X光：雙側肺部多顆圓型陰影，大多出現在肺部的上三分之一處；隨著疾病進展可出現圓形陰影聚集成團。

(C)肺功能：合併阻塞(obstructive)與限制型(restrictive)的肺功能障礙

(D)最低暴露濃度：通常≥50μg/m^3。

(E)最短暴露時間：5年(加速型矽肺症則為2年)。

(F)最長潛伏期：無。

B.急性矽肺症

(A)症狀：快速進展的咳嗽以及喘；體重減輕

(B)胸部X光：雙側肺泡浸潤。

(C)肺功能:限制型(restrictive)的肺功能障礙，氣體交換功能喪失

(D)最低暴露濃度：50μg/m^3。

(E)最短暴露時間：3個月。

(F)最長潛伏期：1年。

(2)結晶型二氧化矽暴露相關的肺癌：

最低暴露濃度：50μg/m^3。

最短暴露時間：5年(加速型矽肺症則為2年)。

最長潛伏期：無。

(3)結晶型二氧化矽暴露相關慢性阻塞型肺疾病：

最低暴露濃度：未知，可能低於50μg/m^3。

最短暴露時間：5年(加速型矽肺症則為2年)。

最長潛伏期：無。

(4)煤礦暴露相關慢性阻塞型肺疾病：

最低暴露濃度：每年100 mg/m^{3}以上。

最短暴露時間：5年。

最長潛伏期：無。

(5)鐵或氧化鐵暴露相關的塵肺症

最低暴露濃度：未知。

最短暴露時間：10年(非常高濃度則為3年)。

最長潛伏期：無。

3.職業性暴露於可呼吸性粉塵(矽)、煤塵或電焊的暴露史。

4.合理的排除了其他原因。

(二)輔助基準

1.肺切片病理檢查：肺部纖維化且有礦物性粉塵微粒。

2.肺部電腦斷層檢查：肺纖維化、毛玻璃變化。

3.生物偵測：電焊工人塵肺症可檢查儲鐵蛋白。

4.相同工作場所或工作內容之其他同事在工作中或之後也產生相同疾病。

參考文獻

[1] International Labour Office. Encyclopedia of occupational health and safety. Vol 11. Geneva: ILO, 1976:1085-1092.

[2] Industrial Injuries Advisory Council. Pneumoconiosis and byssinosis. London: HMSO, 1973.

[3] The Fourth International Pneumoconiosis Conference. Working party on the definition of pneumoconiosis report. Geneva, 1971.

[4] 內政部.粉塵危害預防標準.民70年。

[5] 楊思標.塵肺症--在台灣最初的6例臨床報告。台灣醫誌1952；51: 325-331 。

[6] 楊思標，楊雪舫，陳芳武，王光柱。金瓜石金銅礦山之硅肺症調查研究。台灣醫誌 1953;53:443-454。

[7] 李宣果，徐遐。金銅礦務局風鑽工矽肺症之現狀與建議.國防醫學論文集 1954:59-63。

[8] 李宣果，徐遐，唐謙謀。金瓜石風鑽工矽肺症職業病之再探討，國防醫學論文集 1959:43-45 。

[9] Ko YC, Lai CL. An observation on the deaths of male adults in Juei-Fang from the standpoint of occupational health. 台灣醫誌 1961;60: 76-81.

[10] Ko YC. Silicosis in gold miners: a follow-up study. J Occup Med 1963; 5:43-47.

[11] 黃煥清.從職業衛生觀點對瑞芳鎮成人死亡之一觀察。台灣醫誌1968; 67:522-553 。

[12] 賴信治.某地方金屬礦工之矽肺症的追蹤調查.台灣醫誌1970; 70:604-605。

[13] 吳始明，呂良岳。職業病--塵肺症調查分析研究.台北市社會局，衛生局. 1973。

[14] 馮兆康，林宜長，毛義方。鑄鐵作業勞工暴露游離二氧化矽危害之健康危害研究.國立陽明醫學院公共衛生學研究所碩士論文.1991 。

[15] 朱永釗，張忠孝，呂良岳。台北市塵肺症調查研究.中華醫誌1982;29:227-233。

[16] 施文儀.本省煤礦工人塵肺症防治的情形報告。台灣省政府衛生處.塵肺症防治對策及本省煤礦工人罹患情形報告. 1988:15-23 。

[17] 陳育民，李培元，葉本芳，蘇維鈞，彭瑞鵬。石棉肺症:病例報告與文獻整理.中華醫誌 1990;46:381-384 。

[18] Wang TH, Hwang JJ, Chong IW, Sheen EE, Liu CS, Chen CY. Asbestosis -- report of a case.胸腔醫訊 1990;5:88-89.

[19] 吳仁光，楊泮池，郭壽雄，陸坤泰。肺石綿沉著症--病例報告.內科學誌 1992;3:195。

[20] Xie LZ, Zhou L, Ding BM, Shen H, Han L, Zhou P, et al. Analysis of occupational pneumoconiosis in Jiangsu province from 2006 to 2017. Zhonghua Lao Dong Wei Sheng Zhi Ye Bing Za Zhi. 2019;37(3):189-193.

[21] Ziskind M, Jones RN, Weil1 H. Silicosis. Am Rev Respir Dis 1976;113:643-665.

[22] Graham WGB. Silicosis. Clin Chest Med 1992;13:253-267.

[23] Davis GS. Pathogenesis of silicosis: current concepts and hypotheses. Lung 1986;164:139-154.

[24] Silicosis and Silicate Disease Committee. Diseases associated with exposure to silica and nonfibrous silicate minerals. Arch Pathol Lab Med 1988;112:673-720.

[25] Information notices on occupational diseases: a guide to diagnosis. European Commission Directorate-General for Employment, Social Affairs and Equal Opportunities F4 unit Manuscript completed in January 2009.

[26] IARC, Monographs on the evaluation of the carcinogenic risk of chemicals to humans. Silca, some silicates, coal dust and para-aramid fibrils. 1997, Monograph 68, Lyon: IARC

[27] Leung CC, Yu IT, Chen W. Silicosis. The Lancet 2012, 379, 2008 – 2018.

[28] Seaton A, Legge JS, Henderson J, Kerr KM. Accelerated silicosis in Scottish stonemasons. Lancet 1991;337:341-344.

[29] Snider DE, The relationship between tuberculosis and silicosis. Am Rev Respir Dis 1978;118:455-460.

[30] Morgan EJ. Silicosis and tuberculosis. Chest 1979;75:202-203.

[31] Kleinerman J, Green F, Harley RA, et al. Pathology standard for coal workers' pneumoconiosis. Arch Pathol Lab Med 1979;103:375-433.

[32] Lapp NL, Parker JE. Coal workers' pneumoconiosis. Clin Chest Med 1992;13:243-252.

[33] Morgan WKC, Lapp NL. Respiratory disease in coal miners. Am Rev Respir Dis 1976;113:531-559.

[34] Seaton A. Coal and the lung. Thorax 1983:38:241-243.

[35] Rom WN, Kanner RE, Renzetti AD et al. Respiratory diseases in Utah coal miners. Am Rev Respir Dis 1981;123:372-377.

[36] Wagner JC, Wusteman FS, Edwards JH, et al. The composition of massive lesions in coal miners. Thorax 1975;30:382-388.

[37] Morgan WKC. lndustria1 bronchitis. Br J Ind Med 1978;35: 285-291.

[38] Kibelstis JA, Morgan EJ, Reger R, Lapp NL, Seaton A, Morgan WKC. Preva1ence of bronchitis and airway obstruction in American bituminous coa1 miners. Am Rev Respir Dis 1973;108:886-893.

[39] Ruckley VA, Gauld SJ, Chapman JS, et a1. Emphysema and dust exposure in a group of coal workers. Am Rev Respir Dis 1984;129:528-532.

[40] Hankinson JL, Pa1mes ED, Lapp NL. Pu1monary air space size in coa1 miners. Am Rev Respir Dis 1979;119:391-397.

[41] Morgan WKC, Burgess DB, Lapp NL, Seaton A, Reger RB. Hyperinf1ation of the lungs in coal miners. Thorax 1971;26:585-590.

[42] Perret JL, Plush B, Lachapelle P, Hinks TS, Walter C, Clarke P, et al. Coal mine dust lung disease in the modern era. Respirology. 2017 May;22(4):662-670.

[43] Cosgrove MP. Pulmonary fibrosis and exposure to steel welding fume. Occup Med (Lond). 2015 Dec;65(9):706-12.

[44] Buerke U, Schneider J, Rösler J, Woitowitz HJ. Interstitial pulmonary fibrosis

after severe exposure to welding fumes. Am J Ind Med 2002;41:259–268.
[45] M J Doherty, M Healy, S G Richardson, N C Fisher. Total body iron overload in welder's siderosis. Occup Environ Med 2004;61:82–85.
[46] United States., & National Institute for Occupational Safety and Health. (2007). NIOSH pocket guide to chemical hazards. Cincinnati, Ohio: Dept. of Health and Human Services, Centers for Disease Control and Prevention, National Institute for Occupational Safety and Health.
[47] Parks WR. Occupational lung disorders, 2nd ed. London: Butterworths, 1982.
[48] 行政院勞委會勞工安全衛生研究所。結晶型游離二氧化矽健康危害預防手冊。2008。
[49] International Labour Office: Guidelines for the use of lLO International classification of radiographs of pneumoconiosis. Geneva: International Labour Office, **2011**.
[50] Wu MC. Pu1monary fuoction study in si1icotics. Part 1.spirometric study. 台灣醫誌 1967;66:240-255.
[51] Wu MC. Pulmonary function study in si1icotics. Part 2. arteria1 blood gas study. 台灣醫誌 1967;66:393-409.
[52] 謝遠清，汪大衛，沈建業，江啓輝。煤礦工肺塵症之瀰散量血液氣體分析及通氣功能之研究。中華醫誌 1981;37:103-116。
[53] Merget R, Bauer T, Küpper HU, et al. Health hazards due to the inhalation of amorphous silica. Arch Toxicol 2002; 75: 625–34.
[54] Dreessen WC, Da11ava11e JM, Edwards TL, Sayers RR. Publ Hlth Bull, Wash., No.250, 1940.
[55] Theriault GP, Burgess WA. DiBerardinis LJ, Peters JM. Dust exposure in the Vermont granite sheds. Arch Environ Health 1974;28:12.
[56] Jacobsen M, Rae S, Walton WH, Rogan JM. The relation between pneumoconiosis and dust-exposure in British coal mines. In Inhaled Particles III. edited by Walton WH, Woking: Unwin, 1971:903-917.
[57] Walton WH. Dodgson J, Haddon GG, Jacobsen M. The effect of quartz and

other non-coal dusts in coal workers' pneumoconiosis. Part I: epidemiological studies. In Inhaled Particles IV, edited by Walton WH, Oxford: Pergamon Press,1977:669-700.
[58] Rossiter CE. Relation between content and compositio of coal worker's lung and radiological appearances. Br J Ind Med 1972;29:31-44.
[59] Rossiter CE. Evidence of dose-response relation in pneumoconiosis. Trans Soc Occup Med 1972;22:83-97.
[60] Rossiter CE. Relation of lung dust content to radiological changes in coal workers. Ann N Y Acad Sci 1972;200:465-477 .
[61] Kurth L, Halldin C, Laney AS, Blackley DJ. Causes of death among Federal Black Lung Benefits Program beneficiaries enrolled in Medicare, 1999-2016. Am J Ind Med. 2020 Nov;63(11):973–9.
[62] Almberg KS, Halldin CN, Blackley DJ, Laney AS, Storey E, Rose CS, et al. Progressive Massive Fibrosis Resurgence Identified in U.S. Coal Miners Filing for Black Lung Benefits, 1970-2016. Ann Am Thorac Soc. 2018 Dec;15(12):1420–6.
[63] Kurth L, Casey M, Schleiff P, Halldin C, Mazurek J, Blackley D. Medicare Claims Paid by the Federal Black Lung Benefits Program: US Medicare Beneficiaries, 1999 to 2016. J Occup Environ Med. 2019 Dec;61(12):e510–5.
[64] Yoshii C, Matsuyama T, Takazawa A, Ito T, Yatera K, Hayashi T, et al. Welder's pneumoconiosis: diagnostic usefulness of high-resolution computed tomography and ferritin determinations in bronchoalveolar lavage fluid. Intern Med. 2002 Dec;41(12):1111-7.
[65] Doig AT, McLaughlin ALG. X-ray appearances of the lungs of electric arc welders. Lancet 1936; 1:771–775.
[66] Doig AT, McLaughlin ALG. Clearing of x-ray shadows in welder's siderosis. Lancet 1948; 1:789-91.
[67] Morgan WKC, Kerr HD. Pathologic and physiologic studies of welder's siderosis. Ann Intern Med 1963;55:293-304.

[68] Funahashi A, Schlueter DP, Pintar K, Bemis EL, Siegesmund KA. Welders' pneumoconiosis: tissue elemental microanalysis by energy dispersive x ray analysis. Br J Ind Med. 1988 Jan;45(1):14-8.

[69] Liou SH, TL Gu, SW Hsu, CL Lin, LM Chen. Occupational Illness in Taiwan in Recent Fifty Years: Chemical Hazards-Occupational Lung Diseases. Journal of Medical Sciences. 1992 Jun; 12(6):376-391.

[70] 劉翠溶。塵肺在臺灣和中國大陸發生的情況及其意涵。臺灣史研究 2000；17(4): 113-163。

[71] 李仁智，魏昇堂，施文儀．塵肺症防治對策及本省煤礦罹患情形報告。台灣省衛生處.1988。

[72] 楊錫欽，李統立，楊思標，盧國輝。罹患塵肺症之離職退休煤礦工的族群特徵。中華職業醫學雜誌 2000;7: 123-128。

[73] 行政院勞委會勞工安全衛生研究所。台灣地區塵肺症勞工世代追蹤暨健檢分析研究。1999。

[74] 勞動部勞動及職業安全衛生研究所。石材製品製造業二氧化矽粉塵暴露危害調查研究。2013。

[75] Lin, MH, Su, YC, Tsai, PJ, Hsu , JH. Exposure to Free Silica and Health Effect for Incinerator Maintaining Workers. Chinese J Occup Med. 2010. 17(4)：253-260.

[76] Workers' Hazardous Exposure Survey in Ceramic Manufacturing Industry. In: Institute of Labor, Occupational Safety and Health, Ministry of Labor, R.O.C.; 2013. Report No.: IOSH102-A301

[77] Tsao YC, Liu SH, Tzeng IS, Hsieh TH, Chen JY, Luo JCJ. Do sanitary ceramic workers have a worse presentation of chest radiographs or pulmonary function tests than other ceramic workers? J Formos Med Assoc. 2017 Mar;116(3):139-144.

[78] 索任。病例分享-急性矽肺症。防癆雜誌 2019；秋季號：9-12。
